# Election of Vice-Chairman and Officers

**Published:** 1915-01-23

**Authors:** 


### THE HOSPITAL SUNDAY FUND.

### Election of Vice-Chairman and Officers.

A MEETING of the Council of the Hospital Sunday Fund was held at the Mansion House on Wednesday last, under the presidency of the Right Hon. the Lord Mayor, Sir Charles Johnson.

The Secretary, Mr. Arnold James, read the notice convening the meeting, and the minutes of the previous Council meeting were read and duly approved.

The Secretary said he regretted to have to report to the Council the death of Mr. Thomas Bryant, F.R.C.S., who had been a member of the Council twenty-two years. Sir Ernest Tritton, Bart., moved that the Secretary be instructed to send a letter expressing the Council's regret and condolence with the members of his family. Sir Henry Burdett seconded, and it was carried.

THE DISTRIBUTION COMMITTEE.

Mr. John H. Buxton proposed that the gentlemen whose names already appeared on the Distribution Committee be re-elected, with the addition of the name of the Rev. Hardy Harwood. He felt sure that all present would acknowledge what a valuable addition that gentleman would be. Mr. Harwood had worked at the matter so well that he would be sure to give good sound counsel upon it. The work of the Distribution Committee was of a very responsible nature, and deep thanks were due to them for all they did.

The resolution was carried.

GENERAL PURPOSES COMMITTEE.

Sir Ernest Tritton said he had been requested by the Chairman of the Committee, Prebendary Anderson, who was absent, he regretted to say, through illness, to ask the proposer to submit to the Council for addition to this Committee the name of the Rev. A. J. Hall, Vicar of Holy Trinity, Eltham.

Mr. F. A. Bevan proposed the re-election of the General Purposes Committee, with the addition of the name of the Rev. A. J. Hall, of Eltham, which was carried.

Sir Ernest Tritton, as Chairman of the Committee, asked for the addition of the name of Mr. John Robarts, who was willing to serve upon it. Sir Henry Burdett proposed the re-election of the Committee, with the addition of Mr. Robarts. Prebendary Ingram seconded, and it was carried.

THE NEW VICE-CHAIRMAN.

The Ven. the Archdeacon of London proposed that the Vice-Chairman of the Fund be Sir Ernest Tritton, Bart. He thought the Vice-Chairman required two gifts: to know, and to be known. Sir Ernest knew the Fund's finance from beginning to end; he knew the Committee, the office work, and the constituent subscribers. Obviously many questions behind the scenes must come up between the office and the subscribers, and it would be a great acquisition to have a Vice-

Chairman who really did know the intricacies of the working, so that when it was necessary to consult him it would be felt that he would give sound advice. And Sir Ernest was also known, and he could afford, and was willing to give, the necessary time to the Fund.

Sir Savile Crossley, who seconded, remarked that Sir Ernest Tritton had been for a long time connected with the King's Fund, and there was no one better fitted for the Vice-Chairmanship of this Fund. His election was carried.

Sir Ernest Tritton, in expressing his thanks, said that he had been serving *ad interim* for the last six months, and it had been a pleasure to work with Mr. James and Mr. Lander at the office, where the business progressed quite smoothly.

Election of Honorary Secretaries.

The Rev. Preb. Ingram proposed that the Hon. Secretaries, Sir R. Biddulph Martin and Mr. John H. Buxton, be re-elected. Those gentlemen had been associated with the Fund for many years, and they possessed every qualification. Sir Henry Burdett seconded, and it was carried.

Election of Honorary Auditors.

Mr. F. A. Bevan proposed the re-election of Messrs. Pannell and Co. as hon. auditors. That firm had been for many years associated with the Fund, and had given voluntarily of their services. The Rev. Hardy Harwood seconded, and it was carried.

The Right Hon. Lord Cheylesmore proposed that a hearty vote of thanks be tendered to the Lord Mayor for having presided at the meeting, which was unanimously carried.

The Lord Mayor, in expressing his thanks, said that while he remained Lord Mayor he would have pleasure in welcoming the gentlemen connected with this Fund to the Mansion House, and he would certainly do everything in his power to assist the movement, which was one of the most excellent in the country.

